# Atypical Cardiac Location of Melanoma of Unknown Origin

**DOI:** 10.3390/medicina57020107

**Published:** 2021-01-25

**Authors:** Agnieszka Styczeń, Mariusz Kozak, Marta Karaś-Głodek, Elżbieta Czekajska-Chehab, Andrzej Tomaszewski, Andrzej Wysokiński, Tomasz Zapolski

**Affiliations:** 1 Department of Cardiology, Medical University of Lublin, Jaczewskiego 8, 20-954 Lublin, Poland; styczen.agnieszka@gmail.com (A.S.); mariuszkozak.md@gmail.com (M.K.); karasmarta@o2.pl (M.K.-G.); ajtom@wp.pl (A.T.); a.wysokinski@umlub.pl (A.W.); 2 Department of Radiology, Medical University of Lublin, Jaczewskiego 8, 20-954 Lublin, Poland; czekajska@gazeta.pl

**Keywords:** 64-slice multidetector computed tomography, echocardiogram, melanoma malignum, pembrolizumab

## Abstract

The subject was a 66-year-old woman, suffering from the chest pain evoked by physical activity. Transthoracic echocardiography (TTE) revealed an abnormal structure, 41 × 29 mm. In MSCT, a hypodensic mobile tissue lesion that was infiltrating the whole thickness of left ventricle was confirmed. PET excluded the existence of other remote lesions. After surgical tumor removal, histopathological differential diagnosis revealed melanoma, myoepithelial cancer, and MPNST “high–grade” sarcoma. A control TTE detected a tumor that was 14 × 10 mm. After immunohistochemical results, immunotherapy with pembrolizumab was used, which resulted in complete tumor resolution. Presently, surgical resection and neoadjuvant targeted immunochemotherapy remain the treatment of choice for clinical stage III/IV melanoma.

## 1. Introduction

Primary cardiac tumors are rare [1], and their prevalence is assessed at 0.001 to 0.03% in autopsy series [2]. Metastatic cardiac tumors are far more frequent—from approximately 30- to 40-fold more than primary tumors of the heart [3]. Melanoma malignum is a highly metastatic neoplasm, and the majority of the patients develops remote metastases as a result of neoplastic progression. The most common localizations of the metastases are the liver, bones, and the brain [4]. Malignant melanoma is responsible for 4.4% of cardiac metastases [5]. The median survival for patients with metastatic melanoma is just a few months [6]. The ante mortem diagnosis of metastatic melanoma with heart involvement seems to be an arduous process as less than 16% of patients with this particular neoplasm present with cardiac symptoms [7]. Based on the stage of the disease, therapeutic options include surgery alone (early stage melanomas) or combined with radiotherapy, immunotherapy, chemotherapy, or targeted therapy at advanced stages of the disease [8].

Thereinafter, an interesting case of a 66-year-old female patient with melanoma of an unknown origin with solely heart metastases is presented.

## 2. Case Description

A 66-year-old woman with no history of cardiovascular disease was admitted to the Department of Cardiology of the Medical University of Lublin, on 15th January 2019, because of impaired exercise tolerance, retrosternal chest pain accompanied by numbness of the left upper limb, and temporary heart palpitations. She had experienced the symptoms for several months prior. ECG performed on admission showed the sinus rhythm of 72 bpm, intermediate heart axis, no ST-T segment changes, and no arrhythmias or conduction disorders. The results of basic laboratory tests were within the acceptable range.

**Figure 1:**
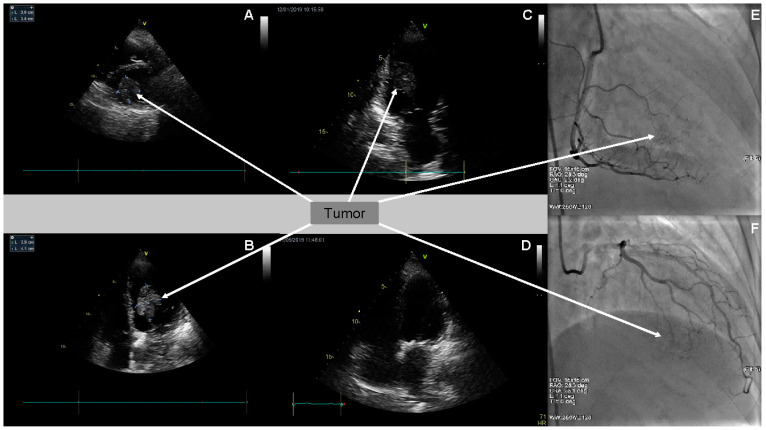
Coronary angiography (CA) and transthoracic echocardiography (TTE). (**A**) CA (RAO 28.3°, CAU 2.2° presentation)—visible poor tumor vascularity at the late arterial phase of the right coronary artery selective angiography (arrow); (**B**) CA (RAO 26.3°, CRA 23.1° presentation)—visible poor tumor vascularity at the late arterial phase of the left coronary artery (circumflex branch) selective angiography (arrow). (**C**) Two-dimensional TTE (parasternal, short axis presentation)—tumor in the left ventricle cavity (arrow); (**D**) 2D TTE (apical 4-Ch presentation)—tumor in the left ventricle cavity (arrow); (**E**) 2D TTE (2-Ch presentation)—tumor in the left ventricle cavity (arrow); and (**F**) 2D TTE (4-Ch presentation)—tumor resolution after chemotherapy.

Two-dimensional transthoracic echocardiography (2D TTE) detected the presence of a large left ventricular mass, 3.0 × 3.4 cm in diameter, in the parasternal short axis presentation, and of 2.9 × 4.1 cm in four chamber (4-Ch) apical presentation (Figure 1A–C). Moreover, it confirmed moderate mitral regurgitation, small tricuspid regurgitation, and small aortic regurgitation. The dimensions of her heart cavities were normal, apart from enlarged left atrium dimension (LAd—4.5 cm). Global left ventricle contractility was preserved—LVEF assessed as 62%. Simultaneously, transesophageal echocardiography (TEE) assessed the tumor originating from and most probably infiltrating the inferior wall of the left ventricle, with singular fibrosis. The diagnosis suggested, on the basis of the TEE image, was left ventricular myxoma.

Due to retrosternal chest pain reported by the patient, coronary angiography (CA) was performed. The angiographic examination excluded significant changes in epicardial coronary arteries. Poor tumor vascularity at the late arterial phase of right coronary artery and circumflex branch of the left coronary artery selective angiography were visualized (Figure 1E,F).

**Figure 2:**
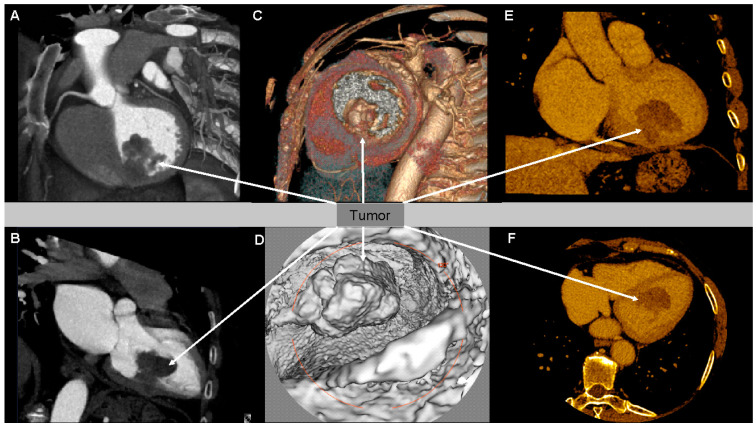
Multi-Slice Computed Tomography (MSCT) showing a pathological tumor mass connected with the posterior wall of left ventricle (LV) (arrows). (**A**) A 3D black-and-white reconstruction with the cutoff plane through the LV long axis; (**B**) a 3D black-and-white reconstruction with the cutoff plane through the LV short axis; (**C**) a 3D volume rendering reconstruction with the cutoff plane through the LV short axis; (**D**) virtual ventriculography of the LV in the navigator option axis; (**E**) a coronal multiplanar reconstruction with gold color scale; (**F**) an axial multiplanar reconstruction with gold color scale.

Subsequently, cardiac CT was performed, confirming the existence of left-ventricle muscle thickening in the area of the knobby lesion. CT scans affirmed a hypodense mobile tissue lesion of irregular outlines originating in the left ventricle wall in medial and the inferoseptal segment, infiltrating the whole thickness of the left ventricle (Figure 2A–F). The dimensions of the lesion were as follows: 45 × 36 × 30 mm. The tumorous mass constricted (infiltration could not be excluded) the posterior papillary muscle. In parenchymal phase, a slight contrast enhancement of the tumor was observed in CT. Tumor vasculature originated most likely mainly from the posterior descending artery (PDA). Segmental contractility abnormalities were not observed in CT, apart from the area neighboring the tumor.

The patient’s case was discussed with and assessed by the Heart Team. On 29 January 2019, the 66-year-old woman was transported to the National Institute of Cardiology in Warsaw, Poland, for further diagnostics and treatment. On admission to the National Institute of Cardiology in Warsaw, cardiac CT was repeated which revealed soft-tissue masses in mid-inferoseptal and inferior segments and stemming from the left ventricle muscle. They constituted a starting point of a large soft-tissue mass measuring 50 × 30 × 25 mm, connected with the inferoseptal LV wall. It was probable that the infiltration of the LV was more advanced than that observed and may have comprised almost the whole inferoseptal and midseptal segments, as well as mid inferior and apical ones. The tumor was balloting and moving towards the mitral valve and the anterior papillary muscle when the patient was in the supine position. The morphology of the mass could be characterized as multiform and having numerous tabs. The process of angiogenesis within the lesion could not be excluded.

On 2 February 2019, in the Department of the Cardiac Surgery and Heart Transplantation of the National Institute of Cardiology in Warsaw, the resection of left ventricle tumor was performed. Because of the size of the lesion, there was no possibility of removing it via mitral valve leaflets. An anterior leaflet was mobilized by removing it from the annulus. Additionally, the mitral valve was repaired with a C-E Physio ring implanted.

A control postoperative TTE showed enlarged left atrium dimension; the dimensions of the remaining heart cavities were normal. There were no segmental contractility disorders. EF was ~60%. In the mitral position, the shadow of the annulus was visible. TTE confirmed small tricuspid regurgitation.

The postoperative course was uneventful. The wound healed properly; the sternum was stable. The patient was transported to the Department of Cardiology of the Medical University of Lublin in a good clinical condition. On the first day after the admission, an episode of AF was registered; the reversion to sinus rhythm was spontaneous. Heart rhythm disturbances did not occur later during the hospitalization, in the Department of Cardiology of the Medical University of Lublin. Furthermore, a PET/CT scan was performed (14 February 2019); the examination revealed an isolated, residual left ventricular tumor, 20 × 16 mm in size. Subsequently, head CT was performed, which revealed neither focal changes nor pathological contrast enhancement areas or intracerebral hemorrhage.

The consecutive TTE showed that the lesion that was 14 × 10 mm in size, in the medial inferior wall ostium, had a different echogenicity. Moreover, it confirmed small tricuspid regurgitation. The TTE image was similar to that performed shortly after the surgery in the Department of Cardiac Surgery and Heart Transplantation of the National Institute of Cardiology in Warsaw. Thereafter, the woman was consulted by the oncologist who proposed further molecular diagnostics aiming at histopathological confirmation and considering a revision cardiac surgery—undertaking the radical treatment. The patient was discharged from the Department of Cardiology in Lublin in a good clinical condition on 1 March 2019. Recommendations for the patient included oral anticoagulation with VKA (warfarin) for a minimum three months, so as to maintain the INR within the target therapeutic range (2.5–3.0).

The histopathological-examination hematoxylin–eosin (H&E) staining on 22 March 2019 revealed loosely lying fragments of fusiform—like transformed cells of neoplasm of a moderate cytologic atypia and a high cellularity. The interstitium was not abundant, having inflammatory infiltrations of chronic type (lymphocytes, plasmocytes). A neoplastic embolus was visible in the vessel’s lumen.

Moreover, IHC staining was performed, and it confirmed the expression of S100 marker in almost all examined tumor cells, as well as the expression of SOX10 marker in the vast majority of neoplastic cells. In FISH examinations, there were no MDM2 gene rearrangement (LDF19/211), SS18 (LDF19/229), and EWSR1 (LDF19/250), thus excluding, accordingly, “intimal sarcoma”, “sarcoma synoviale”, and “clear cell sarcoma”.

A pathomorphologist in the final conclusion suggested, taking into account the sarcomatoid melanoma (metastasis) and “MPNST, high-grade”, based on positive sox10 and s100 positive reactions. The lack of heavy cytokeratins and p40 expression spoke against myoepithelial cancer. Furthermore, the examinations were performed, aiming at indicating the driver mutation. The mutation, which has been confirmed, was the B-RAF mutation, whereas the results for NRAS and NF-1 mutations were negative.

A follow-up TTE carried out on 30 March 2019, in an outpatient clinic, revealed an enlarged left atrium. The global contractility of the left ventricle was slightly impaired (LVEF ~52%). The presence of a left ventricular mass, which was 30 mm × 20 mm in size, related to its inferior wall, was confirmed. The mobility of the mitral valve posterior leaflet was reduced, exhibiting the traits of moderate mitral stenosis. New segmental contractility abnormalities appeared—the hypokinesis of inferior wall and of interventricular septum. In addition, impaired left ventricular relaxation appeared. Small tricuspid regurgitation and small aortic regurgitation were affirmed.

On the basis of immunohistochemical examination result and due to the follow-up TTE confirming the tumor recurrence, the patient was urgently referred to the National Institute of Oncology in Warsaw. Considering the melanoma of an unknown origin with heart metastases as the most probable diagnosis, the intravenous treatment with Keytruda (pembrolizumab) was implemented. Pembrolizumab constitutes a potent, highly selective, fully humanized immunoglobulin (Ig) G4-kappa monoclonal antibody against PD-1 with potential immune checkpoint inhibitory and antineoplastic activities. Keytruda is used in patients with advanced melanoma and is now a standard of care in the first-line setting. Planned treatment consisted of intravenous pembrolizumab 10 mg/kg every three weeks. After the first pembrolizumab infusion, the patient was discharged and went home in a good clinical condition. Subsequent follow-up echocardiography performed on 1 May 2019 revealed no left ventricular tumor (Figure 1D). Moreover, it revealed an enlarged left atrium, moderate mitral stenosis, and small aortic and mitral regurgitation. Tricuspid regurgitation progressed to moderate. Modest interventricular septum hypokinesis was observed. Impaired left ventricular relaxation was confirmed.

The patient underwent subsequent Keytruda intravenous infusions with good tolerance of the treatment. During a successive hospitalization in the National Institute of Oncology in Warsaw, she presented the symptoms of severe vertigo and an episode of faintness. Head MRI detected an acute cerebellar vermis stroke. After clinical state stabilization, the patient was transported to the Neurology Department of Specialist Hospital in Radom (PL), aiming at her further treatment. There, another follow-up TTE confirmed the findings observed in May 2019 and definitely excluded the recurrence of a tumor.

The patient has undergone all the planned pembrolizumab infusion cycles, so far, with good tolerance. Currently, she remains in a good clinical condition and does not present any cardiac symptoms. The patient goes through regular follow-up examinations, both in the National Institute of Oncology in Warsaw and in the regional outpatient clinic of the Medical University of Lublin. Presently, the origin of melanoma is still unknown, and the recurrence of the tumor has not been observed. The 66-year-old is supposed to remain under cardiological and oncological observation for life.

## 3. Discussion

Cardiac metastases complicate the course of neoplastic diseases [9]. Lately, melanoma has become a foremost health problem in numerous countries. The prevalence of melanoma is increasing worldwide in a much more rapid fashion than any other malignancy. The most crucial feature of melanoma is its distinctly high potential to develop metastases. Nearly 1/3 of melanoma patients develop metastatic disease. The most frequent metastatic sites are the lungs, liver, brain, and bones. Cardiac involvement is scarcely identified in clinical base, in less than 10%; however, it is found in over half of patients at autopsy [9,10,11]. Melanoma accounts for 4.4% of all cardiac metastases [12]. Furthermore, melanoma has the highest prevalence of cardiac metastases amongst malignancies [7,13]. Once metastasized to other organs, melanoma is by definition stage IV with poor survival rates; the five-year survival is 15% to 20% [14].

However, the prognosis for melanoma of unknown origin primary lesion (MUP) is usually better than for stage III/IV metastatic melanoma. This is confirmed by the retrospective analysis of 24 patients with identified MUP by Clerico et al. [15]. It was only 1.4% of all cases out of 1720 patients with melanoma at various stages of the disease. There was a significantly better survival for patients with MUP, as compared to patients with metastatic melanoma known primary (MMKP) in stages III/IV. Several theories have been proposed to explain the pathophysiology of MUP. The most plausible theory is that undetected primary melanoma may regress as a result of the host’s immune response, possibly after metastasis has occurred [15]. Cases of spontaneous regression melanoma are commonly translated by modification of the host’s immune system. The oncolytic activity of some viruses, such as retrovirus, is also taken into account [16]. Nevertheless, it is regression as a phenomenon that occurs as a result of the host immune response that attacks primary melanocytic tumor cells via lymphocytes that determine the process of fibrosis, which is increasingly described [17]. The phenomenon of immune response is already used in clinical practice in primary cutaneous melanoma [17]. The better prognosis for patients with MUP is also very similar to the better survival rate for patients with metastatic melanoma coexisting with vitiligo [18]. This correlation may be consistent with the primary lesion regression hypothesis by the host’s immune system. This is also indicated by the median age of the patients at diagnosis, usually older than those with melanoma, which may correspond to a long period of immune disruption between the host and melanoma.

A diagnosis of cardiac involvement before death is infrequent at less than 16% of patients with melanoma present with cardiac symptoms [7]. Cardiac metastases usually tend to remain silent; however, they can cause (1) mechanical complications caused by the limitation of blood flow through the cardiac chambers; (2) electrical complications due to the destruction of the cardiac conduction system by myocardial infiltrative masses; and (3) embolization of the tumor, mimicking transient ischemic attacks or an acute coronary syndrome. Mainly, it affects the right side of the heart [19], but left-side metastases involving the mitral valve have also been reported [20]. Asymptomatic massive involvement of the myocardium has been termed as “charcoal heart” [21]. When present, the clinical signs and symptoms include fatigue, weakness, pericardial effusion, congestive heart failure, cardiac arrhythmia, superior vena cava syndrome, right-ventricular outflow and inflow obstruction, and transient ischemic attack [7,22,23].

Imaging studies are essential for the diagnosis of cardiac metastasis. Chest X-ray may demonstrate cardiomegaly (“water bottle” sign) from a pericardial effusion [24,25]. Because of its non-invasiveness and inexpensiveness, a transthoracic, two-dimensional echocardiography (TTE) remains the method of choice and the first medical image examination used in detecting heart masses. For many tumors, echocardiography can provide information on the location, size, and mobility of cardiac masses [26,27]. Nevertheless, transesophageal echocardiography (TEE) is preferred to TTE, principally when a cardiosurgical procedure is planned [28]. Further imaging, including CT and MRI, may also provide useful information, and PET reassures high specificity and sensitivity, allowing us to visualize metastases at a comparatively initial stage [29]. Currently, gold standard diagnostic imaging is positron emission tomography-computed tomography, which can be used concomitantly with MRI of the brain and CT of the chest, abdomen, and pelvis [30].

Even in the case of a firm suspicion of metastases in the heart, a differential diagnosis of cardiac masses ought to be carried out. Since neoplastic disease places the patients at a higher than average risk of thrombosis, a cardiac thrombus has to be excluded as the potential reason of a tumor. Other possible causes which need to be taken into account during differential diagnosis constitute pseudotrombi (flow-related changes), anatomic variants, and primary cardiac neoplasms. It is important to keep in mind that metastatic cancer may present as malignant pericardial effusion [31]. The rarest examples of metastases in the heart constitute multiple cardiac metastases from a non-functioning pancreatic neuroendocrine tumor (pNET) [32], squamous cell carcinoma of the bladder presenting as a metastatic right ventricular mass [33], metastasis of a pulmonary undifferentiated pleomorphic sarcoma to the right ventricle [34], and extension of adrenocortical carcinoma into the right atrium [35].

Referring to histopathological examination—to the best of our knowledge—the expression of S100 family proteins relates to melanoma progression and clinicopathology. It is widely recognized that the differential expression of these S100 family proteins is directly associated with the development and progression of melanoma malignum. They can thus be used as an independent prognostic indicator in patients with melanoma, especially in the early stages of disease. SOX10 is one of the recently known immunohistochemical markers appropriable in the diagnosis of tumors arising from melanocytes. A large number of reports regarding very high sensitivity of the SOX10 protein in melanoma may indicate that it is a good marker in diagnosing this neoplasm. As opposed to the S100 marker, SOX10 is not expressed in dendritic cells. Limited positive background effect indicates that SOX10 might be an even better diagnostic marker than an S100 one. Generally speaking, the most common driver mutation that leads to characteristic overactivation in the MAPK pathway (which results in an uncontrollable growth and ultimately leads to malignant melanoma) is the B-RAF mutation. Unfortunately, treatment with BRAF inhibitors has met challenges, as patient responses began to drop due to the development of resistance to these inhibitors which paved the way for development of immunotherapies and other small-molecule-inhibitor approaches to address this.

Treatment with immune checkpoint inhibitors has increased long-term survival outcomes in patients with advanced melanoma to as high as 50%; however, individual response can vary greatly. Because of the fact that IHC staining also showed the expression of PD-1 ligand, the therapy with pembrolizumab was commenced. PD-1 is expressed on the surface of cytotoxic T cells, and its ligands-programmed death ligands (PD-Ls) 1 and 2 are expressed on both tumor and immune cells. The inhibition of interactions between PD-1 and PD-L1/PD-L2 by an anti-PD-1 antibody causes the reactivation of cytotoxic T cells, leading to the recognition and destruction of melanoma cells. Diagnostic immunohistochemical assays of PD-L1 have been approved by the FDA (Food and Drug Administration). Pembrolizumab can be characterized as a therapeutic antibody that selectively binds to and blocks PD-1 located on lymphocytes. Inhibiting PD-1 on the lymphocytes prevents it from binding to ligands that deactivate an immune response, permitting the immune system to target and destroy cancer cells; the same mechanism also allows the immune system to attack the body itself, and checkpoint inhibitors, like pembrolizumab, evoke immune-dysfunction adverse effects as a result. Tumors that have mutations that cause impaired DNA mismatch repair, which frequently results in microsatellite instability, tend to generate miscellaneous mutated proteins that could serve as tumor antigens; pembrolizumab seems to facilitate clearance of any such tumor by the immune system, by preventing the self-checkpoint system from blocking the clearance.

What is vital for the patient’s outcome is an early recognition of cardiac metastases. Both invasiveness of the tumor and its anatomic location constitute the factors influencing the workability of cardiosurgical treatment [29]. The patient’s well-being may be enhanced by a complete resection, as well as a conservative surgery. Systemic treatment with chemotherapy, biotherapy, radiotherapy, and immunotherapy belong to other possible methods of treatment [29]. Nowadays, surgical resection and neoadjuvant targeted immunochemotherapy remain the standard of care for clinical stage III and IV melanoma [4]. Moreover, in the presented case, it was the treatment of choice, giving the chance to extend the patient′s life [36].

According to previous experience and to the favorable long-term survival of our MUP patients, a multidisciplinary approach involving different specialists is mandatory [15]. This was also the case in the presented case, where the comprehensive cooperation of cardiologists, cardiac surgeons, radiologists, nuclear medicine specialists, histopathologists, and clinical oncologists allowed for optimal therapy. The clinical effects confirm the effectiveness of the treatment, which is reaffirmed by the two-year observation period of the patient, who is still in good condition, without recurrence of the disease.

## Data Availability

The data presented in this study are available on request from the corresponding author.
